# The salutary effect of peritoneal dialysis catheters on enhanced recovery among high-risk pediatric patients undergoing the left coronary transfer procedure: a cohort study

**DOI:** 10.1186/s12887-021-02913-8

**Published:** 2021-10-20

**Authors:** Chunrong Wang, Yuefu Wang, Fuxia Yan, Peng Fu, Jun Li, Lijing Yang, Sheng Shi, Jianhui Wang, Yuchen Gao, Sudena Wang, Yu Tian

**Affiliations:** 1 Department of Anesthesiology, Fuwai Hospital, Chinese Academy of Medical Sciences, Peking Union Medical College, 167 Beilishi Road, Xicheng District, Beijing, 100037 China; 2 Department of Anesthesiology and Surgical Critical Care Medicine, Beijing Shijitan Hospital, Capital Medical University, No. 10 Tieyi Rd., Yangfangdian, Haidian District, Beijing, 100038 China; 3 Department of Anesthesiology, Qingdao Fuwai Cardiovascular Hospital, City, Shandong Province, Qingdao, China

**Keywords:** Anomalous left coronary artery, Bland-white-Garland syndrome, Cardiac surgical procedures, Peritoneal Dialysis, Enhanced recovery after surgery

## Abstract

**Background:**

Evidence for peritoneal dialysis catheter (PDC) usage in pediatric patients undergoing surgery for deteriorating cardiac dysfunction is lacking. This investigation explored factors associated with PDC usage and its effectiveness in children with anomalous origin of the left coronary artery from the pulmonary artery (ALCAPA).

**Methods:**

Eighty-four children undergoing left coronary artery transfer were retrospectively recruited. The primary endpoint was the postoperative ratio of the general ward/[intensive care unit (ICU)] length of stay. Univariable and multivariable analyses were fitted to assess factors related most strongly to PDC and the ratio of general ward/ICU length of stay.

**Results:**

Of the 84 patients, 17 (20.2%) underwent postoperative PDC placement. Patients with extreme cardiac dysfunction [left ventricular ejection fraction (LVEF) ≤25%] were much more likely to require a PDC (OR, 9.88; 95% CI, 2.13–45.76; *P* = 0.003). Moreover, univariate analysis indicated that concomitant mitral repair significantly decreased the likelihood of PDC placement (OR, 0.25; 95% CI, 0.07–0.85; *P* = 0.026). In those with cardiac dysfunction (LVEF ≤50%), PDC use was associated with a reduced ratio of ward/ICU length of stay (B, − 1.62; 95% CI, − 2.77– -0.46; *P* = 0.008), as was age ≤ 12 months (B, − 1.57; 95% CI, − 2.88– -0.26; *P* = 0.02). At the 1-year follow-up, cardiac improvement was significantly greater in patients with PDC usage than in those without it (*P* <  0.001), and the number of mitral recoveries was comparable between the groups (64.2% vs. 53.3%, *P* = 0.434).

**Conclusion:**

In cohorts with ALCAPA, PDC placement following surgery may be necessary for patients with extreme cardiac compromise, while concomitant mitral repair can probably reduce their usage rate. PDC is beneficial in conferring an improvement in cardiac and mitral performance. Importantly, after patients are transferred from the ICU, recovery efficiency in the general ward can be enhanced by PDC placement, and hospital discharge can therefore be achieved early, especially for patients younger than 12 months or with LVEF ≤50%.

**Supplementary Information:**

The online version contains supplementary material available at 10.1186/s12887-021-02913-8.

## Background

Peritoneal dialysis catheter (PDC) implantation is a critical approach in the perioperative management used in the majority of pediatric institutions to alleviate acute kidney injury (AKI) and fluid overload [1–3]. Importantly, PDC usage, even early preventive usage, has been associated with positive responses [1, 4–7]. Previous investigations offered a variety of detailed information focusing on PDC use in several specific groups, such as neonates or infants [1, 5, 7], patients undergoing the Norwood procedure [6], and patients undergoing other common forms of cardiac repair [4]. It should also be underscored that low cardiac output syndrome is one of the standard indicators for peritoneal dialysis [7]. However, there are currently no studies regarding PDC use in children born with myocardial ischemia and infarction. Pediatric patients diagnosed with left coronary artery from the pulmonary artery (ALCAPA), also known as Bland-White-Garland syndrome, regularly present with typical myocardial ischemia and accompanying mitral regurgitation; their left cardiac function can be extremely compromised, while their right cardiac function is preserved and even normal.

In addition to preoperative comorbidities and complex surgery, extensive care regarding postoperative complications is a main driver of prolonged intensive care unit (ICU) length of stay after congenital heart surgery, as with ALCAPA. However, the quality of first-phase recovery in the ICU can directly determine whether optimal secondary recovery in the general ward and advanced hospital discharge can be achieved, as a prolonged ICU stay is a causative factor for a longer length of ward stay [8]. The general ward/ICU length of stay ratio, which is considered a surrogate for dynamically assessed recovery efficiency, can therefore be decreased with the use of PDC in the ICU.

In this study, we aimed to explore the effects of PDC placement on the ratio of general ward/ICU length of stay following cardiac repair in pediatric patients with cardiac dysfunction since birth, such as ALCAPA.

## Methods

We retrospectively reviewed the medical health records of pediatric patients with ALCAPA who underwent surgical repair at Fuwai Hospital, Beijing, China, from June 2010 until September 2017. This single study was approved by the ethics committee of Fuwai Hospital; however, no written informed consent was obtained from the patients’ parental guardians on the basis of its retrospective nature.

**Fig. 1:**
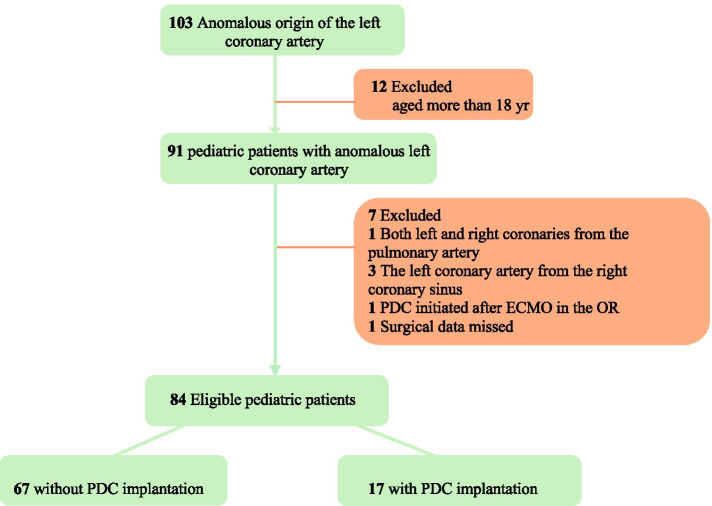
The inclusion and exclusion criteria in the process of recruiting pediatric patients with ALCAPA. PDC, peritoneal dialysis catheter; ECMO, extracorporeal membrane oxygenation; OR, operating room; ALCAPA, anomalous origin of left coronary artery from the pulmonary artery

The participants’ relevant medical data were manually extracted from the institute’s digital record. Patient information was prospectively and comprehensively input by our staff, who were collaboratively in charge of the patients’ entire hospital course. Our analysis included patients with an anomalous origin of the left coronary artery who underwent cardiac surgery (*n* = 103). The exclusion criteria included age ≥ 18 yr, both left and right coronaries extending from the pulmonary artery, left coronary artery from the right coronary sinus, PDC use after extracorporeal membrane oxygenation (ECMO) and no recorded surgical data. The inclusion and exclusion criteria are listed in Fig. 1. A total of 84 pediatric patients were eligible and recruited for the final analysis. Of these 84 patients, one lacked the left main coronary artery but had both left anterior descending and circumflex coronaries derived from the pulmonary artery. Prior to surgery, no patients experienced severe status, as indicated by acute or chronic renal insufficiency, mechanical ventilation support, or hemodynamic support with more than three types of inotropes or ECMO.

### PDC management

Indications for prophylactic PDC insertion in children with ALCAPA were primarily as follows: (1) oliguria or anuria, (2) edema caused by fluid accumulation, (3) electrolyte disturbances (acidosis or hyperkalemia), and (4) high-risk characteristics, including extremely young age (< 6 months), low weight not corresponding to age and severely deteriorating cardiac dysfunction [left ventricular ejection fraction (LVEF) < 20%]. However, whether to prophylactically place a PDC in the operating room was determined by the surgical team, which included cardiac surgeons and anesthesiologists, based primarily on the patient’s risk profile, with the aim of preventing adverse events. After patients were transferred to the ICU, intensivists at the bedside also participated in decision-making regarding PDC placement. The process of catheter placement was carried out by cardiac surgeons. Next, the catheters were either opened to drain or, alternatively, used for dialysis in the ICU. Therapeutic dialysis was initiated for patients who experienced the following events: urine output less than 1 mL/kg/h for 4–8 h, oliguria with poor response to loop diuretics, acidosis or hyperkalemia. Dialysis was performed intermittently at our institute. The indication for PDC removal was confirmed relief of the patient’s critical illness, including urine output normalization, hyperkalemia or acidosis disappearance, and the absence of fluid overload or edema.

### Surgical and intraoperative management

The surgical strategy to correct ALCAPA is reconstruction of the double coronary system. The alternative Takeuchi procedure, usually called intrapulmonary repair, has been abandoned at our institution due to poor surgical outcomes.

It should be mentioned that during cardiopulmonary bypass (CPB), perfusionists carried out modified ultrafiltration for all cohorts. ECMO was initiated if patients had difficulty weaning off CPB, low cardiac output syndrome or severe hemodynamic instability.

Diuretics were administered as first-line therapy to treat suspected AKI. Then, the intensivists and surgeons decided whether to initiate dialysis if patients were unresponsive to high-dose diuretics and the subsequent presence of anuria or oliguria and edema still persisted.

### Clinical definitions

Baseline serum creatinine was determined as the latest value before surgery; this value was not missing for any of the patients. The highest creatinine level on postoperative day (POD) 7 of admission to the ICU was obtained to identify AKI. The pediatric-modified risk, injury, failure, loss and end stage (pRIFLE) criteria, which have been demonstrated to be sensitive in pediatric cohorts, were used to diagnose AKI and categorize patients accordingly. Risk was defined as an estimated glomerular filtration rate (eGFR) that had decreased by 25–49% compared with its baseline value, injury as a decrease of 50–74%, and failure as a decrease ≥75% or an eGFR < 35 mL/min/1.73 m^2^ [9]. Any degree of failure or loss was perceived as severe AKI. The Schwartz model formula was applied to determine the eGFR [10]. Fluid balance between the operative period and POD 3 was calculated, and negative fluid balance was set as a value less than 0%. For patients with PDC placement in the ICU, the timing of the placement was also obtained. Vasoactive-inotropic score (VIS) = dopamine (μg/kg/min) + dobutamine (μg/kg/min) + 100 × epinephrine (μg/kg/min) + 10 × milrinone (μg/kg/min) + 10,000 × vasopressin (U/kg/min) + 100 × norepinephrine (μg/kg/min) [11]. A prolonged duration of mechanical ventilation was defined as more than 100 h because this cutoff is the upper 75% limit of the interquartile range, and 25% of the cohort exceeded this value. Cardiac improvement was the change in LVEF during the interval between the preoperative visit (T1) and hospital discharge or the 1-year follow-up time point (T2). Thus, the change in LVEF was calculated by the following equation: 100 %  ∗ (*LVEF*_(T2)_ − LVEF_(T1)_)/LVEF_(T1)_. The degrees of mitral regurgitation were categorized as none/trivial, mild, moderate and severe. The amelioration of mitral regurgitation at the 1-year follow-up was defined as follows: (a) the classification was degraded for patients diagnosed preoperatively as having mild, moderate or severe regurgitation; and (b) there was no further upgrade or aggressive deterioration in patients with no/trivial regurgitation.

### Outcome of interest

Given that patients with PDC placement recover efficiently in the ICU, they will have a decreased length of stay in the general wards before successful hospital discharge. The ratio of general ward/ICU length of stay was therefore adopted as a primary outcome in the present study.

### Statistical analysis

Variables following a normal distribution are expressed as the mean ± standard deviation. However, a majority of the continuous data had a skewed distribution and were thus presented as the median (interquartile range). Frequencies and percentages were also used, as appropriate, for categorical data. For continuous variables, t-tests or Mann-Whitney U tests were used to assess differences between groups. Comparisons of categorical data were made by the chi-square test in combination with Yate’s continuity correction or Fisher’s exact test.

Backward stepwise multivariate analyses were used to identify the predictors of PDC placement and elucidate the association between PDC placement and the ratio of general ward/ICU length of stay. A *P* value < 0.1 based on the univariate analysis was used to identify variables to be electively entered into the final models. Discrimination performance was assessed using the receiver-operator characteristic area under the curve (AUC-ROC) and the Hosmer-Lemeshow goodness-of-fit test for calibration. Odds ratios (ORs) and B and their associated confidence intervals (CIs) were reported. Analysis of the variance inflation factor was performed to identify the existence of multicollinearity among the included variables.

All statistical analyses were performed using SPSS 24.0 software (IBM, Armonk, NY, USA). Figures were generated with GraphPad Prism 7.0 in conjunction with Microsoft office word 2016.

## Results

Overall, there were 84 eligible patients; the mean age was 12.5 months (7.0–40.5), and half of the patients were younger than 12 months. The proportion of males (58.3%) was slightly greater than that of females. The median weight was 9.0 kg (6.5–14.0). Prior to surgery, almost 50% of patients experienced cardiac dysfunction (LVEF ≤50%). Forty-one patients underwent concomitant mitral repair. Among the entire cohort, 20.2% (17/84) of the children underwent PDC placement.

The patients who underwent PDC insertion were significantly younger (*P* <  0.001) and weighed significantly less (P <  0.001) than those who did not have PDC. Additionally, patients with PDC placement had significantly worse cardiac function; for example, their LVEF values at baseline were significantly decreased (P <  0.001) and their creatinine kinase-MB levels were higher (*P* = 0.001). The number of patients who underwent concomitant mitral repair was significantly lower in the PDC group (*P* = 0.02). Other baseline and operative performance variables are shown in Table 1.

**Table 1:** Demographic and surgical presentation of the included patients

	Total (*n* = 84)	No PDC (*n* = 67)	PDC (*n* = 17)	P
Demographics				
Age at surgery, months, median (IQR)	12.5 (7.0, 40.5)	20.0 (8.0, 55.0)	6 (3, 11.0)	< 0.001
4–12 months, n (%)	30 (35.7)	21 (31.3)	9 (52.9)	
≤ 4 months, n (%)	12 (14.3)	6 (9.0)	6 (35.3)	
Male, n (%)	49 (58.3)	42 (62.7)	7 (41.2)	0.108
Weight, kg, median (IQR)	9.0 (6.5, 14.0)	11.0 (7.7, 16.5)	6.0 (5.65, 7.40)	< 0.001
LVEF, %, median (IQR)	55.0 (25.0, 65.8)	60.0 (37.0, 68.0)	18.0 (15.9, 22.5)	< 0.001
LVEF ≤50%, n (%)	42 (50.0)	23(34.3)	17(100)	< 0.001
LVEF ≤25%, n (%)	23 (27.4)	9 (13.4)	14 (82.4)	
LVEDD, mm, median (IQR)	40.5 (36.3, 45.0)	40.0 (37.0, 45.0)	41.0 (35.5, 46.0)	0.668
Mitral regurgitation, n (%)				0.121
None/trivial	21 (25.0)	16 (23.9)	5(29.4)	
Mild	21 (25.0)	15(22.4)	6(35.3)	
Moderate	20 (23.8)	15(22.4)	5(29.4)	
Severe	22 (26.2)	21(31.3)	1(5.9)	
Serum creatinine, mg/dL, median (IQR)	0.31 (0.26, 0.40)	0.31 (0.27, 0.41)	0.32 (0.22, 0.35)	0.28
Albumin, g/dL, mean (SD)	43.9 ± 3.1	44.0 ± 3.3	43.9 ± 2.4	0.932
CK-MB, U/L, median (IQR)	25.0 (19.3, 35.0)	24.0 (19.0, 33.0)	38.0 (23.0 70.5)	0.001
Therapy prior to surgery, n (%)				
β-blocker	1 (1.2)	1(100)	0 (0)	–
ACEI	6 (7.1)	5 (7.5)	1 (5.9)	1.0
Diuretics	32 (38.1)	21 (31.3)	11 (64.7)	0.011
Digoxin	17 (20.2)	14 (20.9)	3 (17.6)	1.0
Surgical data				
Reconstruction of mitral valve, n (%)	41(48.8)	37 (55.2)	4 (23.5)	0.02
Cardiopulmonary bypass, min, median (IQR)	111.0 (93.3, 138.5)	111.0 (92.0, 139.0)	109.0 (94.5, 142.5)	0.789
Cardiopulmonary bypass ≥120 min, n (%)	32 (38.1)	24 (35.8)	8 (47.1)	0.394
Crossclamp time, min, median (IQR)	69.0 (55.3, 86.0)	70.0 (57.0, 90.0)	67.0 (47.0, 82.0)	0.268

*PDC* peritoneal dialysis catheter; *LVEF* left ventricular ejection fraction; *LVEDD* left ventricular end-diastolic diameter; *CK-MB* creatinine kinase-MB; *ACEI* angiotensin-converting enzyme inhibitors; *IQR* interquartile range; *SD* standard deviation

Postoperative outcomes in patients with and without PDC are shown in Table 2. There was no significant difference in fluid balance within 3 days postoperatively between the groups. However, patients requiring PDC had significantly greater incidences of severe AKI and prolonged duration of mechanical ventilation and higher VIS scores than those without PDC insertion. Over the 1-year follow-up, the improvement in the change in LVEF in the PDC group was significantly better than that in the non-PDC group (120.8% (50.0, 203.4), 6.7% (− 3.1, 44.7), *P* <  0.001), and the proportion of patients in whom mitral regurgitation was alleviated was comparable between the 2 groups (64.2, 53.3%, *P* = 0.434).

**Table 2:** Clinical outcome comparison between the groups following the left coronary transfer procedure

	No PDC (*n* = 67)	PDC (*n* = 17)	P
Variance of fluid balance			
POD 1^a^, n (%)			0.212
Negative	8 (11.9)	2 (12.5)	
0–5%	39 (58.2)	5 (21.3)	
5–10%	15 (22.4)	6 (37.5)	
>10%	5 (7.5)	3 (18.8)	
POD 2 ^a^, n (%)			0.266
Negative	55 (82.1)	10 (62.5)	
0–5%	10 (14.9)	5 (31.3)	
5–10%	2 (3.0)	1 (6.3)	
>10%	0 (0)	0 (0)	
POD 3 ^a^, n (%)			
Negative	46 (68.7)	14 (87.5)	0.227
0–5%	19 (28.4)	2 (12.5)	
5–10%	2 (3.0)	0 (0)	
>10%	0 (0)	0 (0)	
Negative fluid balance by cumulative 3 days, n (%)	20 (29.9)	6 (37.5)	0.553
VIS score, median (IQR)			
1 h	12.0 (8.8, 15.3)	20.5 (17.5, 23.0)	< 0.001
6 h	12.5 (10.0, 18.0)	17.3 (13.3, 20.5)	0.024
12 h	11.5 (8.0, 17.0)	19.0 (12.0, 22.0)	0.012
24 h	12.0 (8.0, 16.0)	22.0 (14.5, 23.4)	0.002
48 h	9.5 (2.0, 13.0)	16.0 (12.3, 17.0)	< 0.001
AKI, n (%)	42 (62.7)	14 (82.4)	0.124
Severe AKI, n (%)	12 (17.9)	9 (52.9)	0.008
Injury	10 (83.3)	8 (88.9)	
Failure	2 (16.7)	1 (11.1)	
Duration of MV, h, median (IQR)	17.0 (8.0, 27.0)	231.0 (97.5, 969.5)	< 0.001
Prolonged MV ≥100 h, n (%)	7 (10.4)	13 (76.5)	< 0.001
Change in LVEF until hospital discharge^b^, median (IQR)	0 (−7.4, 13.5)	49.4 (7.0, 80.7)	0.001
The entire hospital LOS, d, median (IQR)	8.0 (7.0, 11.0)	28.0 (18.0, 91.0)	< 0.001
POD-ICU LOS, d, median (IQR)	2.0 (1.0, 5.0)	20.0 (8.5, 62.0)	< 0.001
POD-general ward LOS, d, median (IQR)^b^	6.0 (5.0, 7.0)	10.0 (7.0, 21.0)	< 0.001
LOS ratio of ward/hospital^b^, median (IQR)	0.71(0.50, 0.86)	0.39 (0.20, 0.50)	< 0.001
LOS ratio of ward/ICU^b^, median (IQR)	2.5 (1.0, 6.0)	0.65 (0.25, 1.0)	< 0.001
1-year follow up			
Change in LVEF^b^, %, median (IQR)	6.7 (−3.1, 44.7)	120.8 (50.0, 203.4)	< 0.001
Improvement of mitral regurgitation^b^, n (%)	43 (64.2)	8 (53.3)	0.434

^a^One child was excluded in the PDC group who underwent catheter placement on POD 7. ^b^Two children were not included in the PDC group because both died in the ICU before their readmission to the ward. *PDC* peritoneal dialysis catheter; *POD* postoperative day; *VIS* vasoactive-inotropic score; *AKI* acute kidney injury; *MV* mechanical ventilation; *LOS* length of stay; *ICU* intensive care unit; *h* hours; *d* days; *LVEF* left ventricular ejection fraction; *IQR* interquartile range; *SD *standard deviation

### Risk factors for PDC placement

An LVEF ≤25% enhanced the odds of PDC placement by nearly 10-fold [OR = 9.88, 95% CI 2.13 to 45.76; *P* = 0.003]. This model had good discrimination (AUC-ROC 0.918) and calibration (Hosmer-Lemeshow statistic *P* = 0.594). Additionally, concomitant mitral repair was associated with lower odds of PDC usage in the univariable analysis (OR = 0.25, 95% CI 0.07 to 0.85, *P* = 0.026) (Table 3).

**Table 3:** Association between risk factors and PDC placement according to univariable and multivariable logistic regression analyses

Variables	Univariate analysis	Multivariate analysis		
	OR (95% CI)	P	OR (95% CI)	P
LVEF ≤25% (yes/no)	30.01 (7.19, 125.8)	< 0.001	9.88 (2.13, 45.76)	0.003
Age ≤ 4 mo (yes/no)	5.55 (1.51, 20.37)	0.01		
Weight (per 1 kg increment) *	0.55 (0.38, 0.79)	0.01	0.66 (0.44, 0.99)	0.045
Concomitant mitral repair (yes/no)	0.25 (0.07, 0.85)	0.026		
CPB ≥120 min (yes/no)	1.59 (0.54, 4.67)	0.396		
Serum creatinine (per 1 mg/dL increment) *	0.1 (0, 4.0)	0.132		
Albumin (per 1 g/dL increment)*	0.99 (0.84, 1.18)	0.931		

*Those covariables were entered into the regression analyses in the form of continuous data. *PDC* peritoneal dialysis catheter; *OR* odds ratio; *CI* confidence interval; *LVEF* left ventricular ejection fraction; *CPB* cardiopulmonary bypass

### Association between PDC and the ratio of ward/ICU length of stay

Compared with patients without PDC, those with PDC placement had significantly extended lengths of stays in the ICU (20.0 days (8.5, 62.0), 2.0 days (1.0 to 5.0), *P* <  0.001) and general ward (10.0 days (7.0, 21.0), 6.0 days (5.0 to 7.0), *P* < 0.001).

The primary outcome, the ratio of ward/ICU length of stay, was significantly lower in patients who underwent PDC implantation [0.65 (0.25 to 1.0), 2.5 (1.0 to 6.0), *P* < 0.001] than in those who did not. An LVEF > 50% (B = 1.82, 95% CI 0.52 to 3.12; *P* = 0.007) and age > 12 months (B = 1.61, 95% CI 0.31 to 2.91; *P* = 0.016) were both significant predictors of an increased ratio of general ward/ICU length of stay. However, the univariable analysis showed that PDC placement was strongly associated with a decreased ratio of general ward/ICU length of stay (B = − 2.88, 95% CI − 4.46 to − 1.31; P < 0.001) (Table 4). The variance inflation factor of this multivariable linear regression was < 2 for all the included covariables; thus, among these variables, there was no possibility for multicollinearity.

**Table 4:** Association between patients’ characteristics and the ratio of general ward/ICU length of stay based on multivariable linear regression analysis

Variables	Univariate analysis	Multivariate analysis		
	B (95% CI)	P	B (95% CI)	P
LVEF > 50% (yes/no)	2.57 (1.38, 3.76)	< 0.001	1.82 (0.52, 3.12)	0.007
Age > 12 mo (yes/no)	0.02 (0.01, 0.04)	0.006	1.61 (0.31, 2.91)	0.016
Weight (per 1 kg increment) *	0.07 (0.01, 0.12)	0.019		
Moderate/severe MR (yes/no)	−0.09 (−1.40, 1.23)	0.894		
CPB (per 1 min increment) *	− 0.01 (− 0.02, 0.00)	0.193		
Hemoglobin (per 1 mg/dL increment) *	0.06 (0.02, 0.10)	0.009		
Serum creatinine (per 1 mg/dL increment) *	4.00 (−1.63, 9.62)	0.161		
Albumin (per 1 g/dL increment) *	−0.02 (−0.23, 0.20)	0.872		
PDC (yes/no)	−2.88 (−4.46, −1.31)	< 0.001		
Severe AKI (yes/no)	−1.21 (− 2.72, 0.29)	0.113		
FO postoperative day 1 (per 1% increment) *	−0.08 (−0.18, 0.02)	0.131		

*Those confounding variables were entered into the regression analyses in the form of continuous data. *ICU* intensive care unit; *CI* confidence interval; *LVEF* left ventricular ejection fraction; *MR* moderate regurgitation; *PDC* peritoneal dialysis catheter; *AKI* acute kidney injury; *FO* fluid overload

### Subgroup analysis

**Fig. 2:**
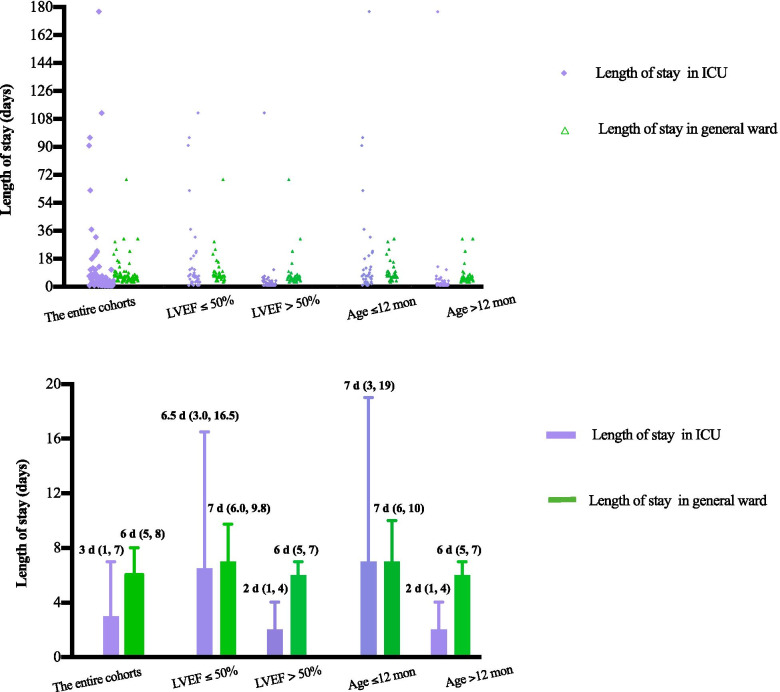
Distribution of the duration of ICU and general ward lengths of stays following the left coronary procedure in the entire cohort, in patients with and without cardiac dysfunction (LVEF ≤50%) and in patients younger and older than 12 months. Two patients with LVEF ≤50% and age ≤ 12 months died in the ICU and were never transferred to the general ward; thus, neither were included in the analyses. The upper panel depicts the scatter distributions among all individuals regarding the length of ICU and ward stays, whereas the lower panel depicts the median (interquartile) days in the ICU and in the ward in each group. The ICU [6.5 days (3.0, 16.5), 2.0 days (1.0, 4.0), *P* < 0.001] and ward [7.0 days (6.0, 9.8), 6.0 days (5.0, 7.0), *P* = 0.023] lengths of stays were significantly prolonged in cohorts with cardiac dysfunction compared with those without cardiac dysfunction. Similarly, compared with older children, both the ICU [7.0 days (3.0, 19.0), 2.0 days (1.0, 4.0), *P* < 0.001] and general ward lengths of stays [7.0 days (6.0, 10.0), 6.0 days (5.0, 7.0), *P* = 0.006] were also significantly longer in patients under 12 months old. ICU, intensive care unit; LVEF, left ventricular ejection fraction

Among the 82 patients surviving after surgery, the proportions of PDC placement in patients ≤12 months and those > 12 months were 32.5% (13/40) and 4.8% (2/42), respectively. Additionally, the incidences of PDC placement in those with LVEF ≤50% and LVEF > 50% were 37.5% (15/40) and 0% (0/42), respectively. The distributions of length of stay in the ICU and general ward categorized by age and LVEF are depicted in Fig. 2. Patients with LVEF > 50% had a significantly higher ratio of ward/ICU length of stay than those with LVEF ≤50% [3.5 (1.42 to 6.0), 0.86 (0.52 to 2.0), *P* < 0.001]. Additionally, compared with patients > 12 months, those ≤12 months had a lower ratio of ward/ICU length of stay [1.07 (0.58 to 2.0), 4.25 (1.25 to 6.0), *P* < 0.001].

Subgroup analyses were performed in surviving pediatric patients with a high-risk profile, namely, those with LVEF ≤50% (*n* = 40) or those younger than 12 months (n = 40) (Supplemental Tables 1 and 2). Remarkably, PDC placement was an independent predictor of a reduced ratio of ward/ICU length of stay in both of these subgroups (B = − 1.62; 95% CI − 2.77 to − 0.46; *P* = 0.008 for LVEF ≤50%; B = − 1.57; 95% CI − 2.88 to − 0.26; *P* = 0.02 for age ≤ 12 months).

### Detailed information on cohorts with PDC placement

The number of patients with PDC placement at the time of surgery completion, within 24 h after transfer to the ICU and after more than 24 h in the ICU was 9, 5 and 3, respectively. Of those 17 patients, the majority (70.6%, 12/17) underwent dialysis in the ICU, whereas the others underwent peritoneal drainage. The serum creatinine level at dialysis initiation was significantly greater than that at baseline (0.64 mg/dl ± 0.22, 0.28 mg/dl ± 0.07, *P* < 0.001). The average LVEF at the time of dialysis was 22.1% ± 6.4.

Of the 2 children who died before ICU discharge, one (preoperative LVEF, 10%) had a PDC placed in the operating room, and the other (preoperative LVEF, 20%) had it placed within 24 h postoperatively. Of the remaining surviving cohort (*n* = 15), at the three time points of baseline, hospital discharge and 1-year follow-up, their cardiac performance significantly increased from 18.0% (16.4, 19.5) to 30.0% (20.8, 55.0) to 45.0% (28.0, 64.0) (P < 0.001).

## Discussion

In patients with ALCAPA who underwent surgical repair, a lower LVEF at baseline was a risk factor for PDC placement, while concomitant mitral repair decreased the likelihood of PDC placement. In patients with a LVEF ≤50% or an age ≤ 12 months, PDC placement had the potential to reduce the ratio of ward/ICU length of stay, thus contributing to fewer days in the general ward and early discharge from the hospital after transfer from the ICU.

Intrinsic cardiac ischemia since birth and its associated consequences contribute to the complex pathology, which determined whether the children in our study underwent the left coronary transfer procedure. By selecting a uniform group with ALCAPA, we minimized the bias associated with the surgical procedure or diversity of illness physiology. Although the standard criteria for PDC placement have not been definitive to date, its use should be determined according to the individual’s surgical complexity [4, 7] and unique pathophysiology [6, 12]. For example, patients undergoing the Norwood procedure needed PDC due to their sensitivity to fluid fluctuation and hemodynamic modification [6].

We chose to have the ratio of ward/ICU length of stay as our primary outcome as a surrogate for recovery efficiency. In contrast to conventional endpoints such as observed incidences of adverse events or medical costs, this ratio can reflect patient recovery in a continuous and dynamic manner throughout the entire hospitalization phase, namely, from transfer from the ICU to the general ward and, ultimately, hospital discharge. In patients with unclear indication for postoperative critical care, ICU admission was probably associated with earlier hospital discharge and lower overall costs due to improved treatment in the ICU [13]. In addition, PDC placement at the time of CPB in infants could decrease the additional cost expenditure within the ICU for respiratory therapy, laboratory procedures, and medications by improving clinical outcomes including earlier extubation, lower inotrope requirements and less time to negative fluid balance [1]. Therefore, PDC placement is salutary in improving recovery quality in the ICU, where higher acuity and complex care are meticulously conferred; thereafter, upon returning to the general ward, fewer residual lesions remain such that patients can meet hospital discharge criteria early and in a healthier state. In our study, PDC usage was a protective factor associated with the ratio of ward/ICU length of stay, specifically in high-risk cohorts with LVEF ≤50% or younger than 12 months. However, this is an exploratory study, and further studies are needed to validate the clinical utility of this novel endpoint.

We did not prefer to use ICU length of stay as the primary outcome. Clearly, following surgery, pediatric cohorts with critical illness generally experience a prolonged length of stay in the ICU [14–16]. A prolonged ICU stay in pediatric cardiac settings has been identified to be a straightforward consequence of difficult cases and vice versa; it can also cause adverse effects, including decreased late survival [14, 15], significant resource costs [14] and bed occupancy shortages [14, 16]. Another study demonstrated that pediatric patients who underwent PDC placement experienced a decreased duration of ICU stay [2]; however, we reached the opposite conclusion. The most likely explanation for this discrepancy is that patients with PDC placement had a high-risk profile and frequently experienced intraoperative critical complications, e.g., worsening cardiac dysfunction, severe AKI, higher requirement of inotropes and extended hours of mechanical ventilation. The benefits of PDC regarding recovery were therefore offset by postoperative complications; additionally, adjunctive dialysis therapy had a role in extending the ICU length of stay.

We additionally demonstrated that concomitant mitral repair could decrease the odds of PDC implantation by 0.75-fold. Whether to perform mitral procedures is still under debate [17–19]. However, in recent decades, mitral repair has been continuously accepted. Early concomitant mitral repair is encouraged because it yields satisfactory outcomes [19], specifically in populations with either severe regurgitation [20] or structural lesions [18]. We conjecture that mitral surgery can confer benefits in hemodynamic recovery within early postoperative episodes; consequently, kidney insult and cardiac dysfunction may be significantly decreased.

Intensivists at our institute actually implemented an optimal fluid-limiting strategy following cardiac surgery for pediatric patients, and two-thirds of the fluid maintenance was administered during POD 1. Kwiatkowski et al. [1] attested that PDC placement was beneficial in achieving both earlier and rapid negative fluid status by POD 1 and 2. However, this type of benefit of PDC placement was not observed in our study, as negative fluid balance may not be achieved within a limited number of days [2, 6]. The proportion of patients with drain placement was 29.4%, which is relatively higher than that in Kwiatkowski’s study [1], where fewer than 10% of patients underwent drain placement by POD 3 [1]. Drainage and dialysis were proposed to have equal effects on negative fluid balance [1].

Cardiorenal syndrome is a lasting concern following congenital cardiac repair. It has been reported that postoperative AKI can develop frequently in patients with anomalous left coronary arteries [21]. Pediatric patients categorized as risk adjustments for congenital heart surgery-3, such as ALCAPA, have a 2-fold higher chance of PDC use [4]. We cannot deny the fact that at our institution, the primary goal of PDC placement is the prevention of kidney disease progression. In the current study, patients with placement of a PDC had greater odds of severe AKI, whereas the serum creatinine level when dialysis was initiated was higher than that at baseline. We did not observe the final effect of PDC placement or dialysis on AKI mitigation; however, in our routine management, the PDC was removed if a patient’s urine output normalized.

The idea of placing a PDC as soon as the CPB procedure is completed has been proposed [1, 2]; nevertheless, this protocol is not widely implemented. The earliest timing for PDC placement by our cardiac surgeons was just after the surgical repair was completed. Notably, PDC placement at the bedside in the ICU will enhance the risks of infection, additional injury, leakage and catheter displacement. In our cohort, 9 patients underwent PDC placement in the operating room, while the remaining 8 underwent PDC placement in the ICU without any associated complications.

### Study limitations

The inherent retrospective characteristics of this study contributed to the predominant limitation. Another shortcoming was that no specific effect of PDC placement on short- and long-term mortality was estimated, as there were only 2 in-hospital deaths. Moreover, the complex association between AKI and PDC implantation cannot be further solved due to chronological uncertainty. After all, more than 60% of PDCs are placed in pediatric patients lacking acute kidney failure [4]. Finally, we did not acquire accurate serum creatinine levels or urine output counts at the time of PDC implantation. Oliguria was diagnosed based on our intensivists’ crude estimation while referring to the standard rule of urine output < 1 mL/kg/h for 4–8 h.

## Conclusions

Extremely severe cardiac dysfunction can increase the prevalence of PDC implantation following surgical repair in patients with ALCAPA. Concomitant mitral repair is of marginal benefit with regard to reducing the likelihood of PDC placement. Among high-risk cohorts, such as those with LVEF ≤50% or age ≤ 12 months, PDC placement can reduce the ratio of general ward/ICU length of stay. Overall, patient recovery efficiency in the general ward can be significantly improved with PDC implantation in the operating room or the ICU, and rapid hospital discharge can therefore be achieved.

## Supplementary Information

**Additional file 1 **

## Data Availability

The datasets used and analyzed in the current study are available from the corresponding authors on reasonable request.

## Abbreviations

PDC: Peritoneal dialysis catheter
AKI: Acute kidney injury
ALCAPA: Left coronary artery from the pulmonary artery
ICU: Intensive care unit
ECMO: Extracorporeal membrane oxygenation
LVEF: Left ventricular ejection fraction
CPB: Cardiopulmonary bypass
POD: Postoperative day
pRIFLE: The pediatric-modified risk, injury, failure, loss and end stage criteria
eGFR: Estimated glomerular filtration rate
VIS: Vasoactive-inotropic score
AUC-ROC: Area under the receiver operating characteristic curve
OR: Odds ratio
CI: Confidence interval
