# Supplementary material for: The salutary effect of peritoneal dialysis catheters on enhanced recovery among high-risk pediatric patients undergoing the left coronary transfer procedure: a cohort study
Source: BMC Pediatr. 2021 Oct 20;21:461. doi: 10.1186/s12887-021-02913-8 (PMC8527684; doi:10.1186/s12887-021-02913-8)
Supplement: Supplementary file 1 — Additional file 1 [file 12887_2021_2913_MOESM1_ESM.docx]

| Supplemental Table 1. Risk factors associated with ratio of ward/ICU length of stay in patients with left ventricular ejection fraction ≤ 50%. | | | | |
| --- | --- | --- | --- | --- |
| Variables | Univariable linear analysis Multivariable linear analysis | | | |
|  | B (95% CI) P B (95% CI) P | | | |
| LVEF > 25% (yes/no) | 1.3 (0.12, 2.48) | 0.032 |  |  |
| Age (per 1 month increment)* | 0.02 (-0.01, 0.06) | 0.220 |  |  |
| Weight (per 1 kg increment)* | 0.24 (0.02, 0.47) | 0.034 |  |  |
| Moderate/severe mitral regurgitation (yes/no) | -0.55 (-1.82, 0.72) | 0.383 |  |  |
| CPB (per 1 min increment)* | -0.01 (-0.02, 0.00) | 0.158 |  |  |
| Hemoglobin (per 1 mg/dL increment)* | 0.01 (-0.05, 0.06) | 0.770 |  |  |
| Serum creatinine (per 1 mg/dL increment) * | -6.55 (-17.8, 4.71) | 0.246 |  |  |
| Albumin (per 1 g/dL increment) * | -0.05 (-0.3, 0.2) | 0.688 |  |  |
| PDC (yes/no) | -1.62 (-2.77, -0.46) | 0.008 | -1.62 (-2.77, -0.46) | 0.008 |
| Severe AKI (yes/no) | -1.01 (-2.29, 0.26) | 0.116 |  |  |
| Fluid balance POD 1 (per 1% increment) * | -0.03 (-0.1, 0.04) | 0.434 |  |  |
| Forty patients were finally recruited after 2 were excluded because of postoperative death in ICU. * Those covariables were presented as continuous data when entering analyses. ICU, intensive care unit; CI, confidence interval; LVEF, left ventricular ejection fraction; CPB, cardiopulmonary bypass; PDC, peritoneal dialysis catheter; AKI, acute kidney injury; POD, postoperative day. | | | | |

| Supplemental Table 2. Risk factors associated with ratio of ward/ICU length of stay in patients ≤ 12 months. | | | | |
| --- | --- | --- | --- | --- |
| Variables | Univariable linear analysis Multivariable linear analysis | | | |
|  | B (95% CI) P B (95% CI) P | | | |
| LVEF > 25% (yes/no) | 1.31 (0.06, 2.56) | 0.040 |  |  |
| Age (per 1 month increment)* | 0.07 (-0.14, 0.29) | 0.496 |  |  |
| Weight (per 1 kg increment)* | 0.15 (-0.28, 0.57) | 0.488 |  |  |
| Moderate/severe mitral regurgitation (yes/no) | 0.09 (-1.24, 1.42) | 0.893 |  |  |
| CPB (per 1 min increment)* | -0.01 (-0.02, 0.00) | 0.225 |  |  |
| Hemoglobin (per 1 mg/dL increment)* | -0.01 (-0.07, 0.06) | 0.857 |  |  |
| Serum creatinine (per 1 mg/dL increment)* | -7.25 (-16.92, 2.41) | 0.137 |  |  |
| Albumin (per 1 g/dL increment)* | -0.21 (-0.41, -0.00) | 0.047 |  |  |
| PDC (yes/no) | -1.57 (-2.88, -0.26) | 0.02 | - 1.57 (-2.88, -0.26) | 0.02 |
| Severe AKI (yes/no) | -0.44 (-1.82, 0.94) | 0.552 |  |  |
| Fluid balance POD 1 (per 1% increment)* | -0.02 (-0.11, 0.07) | 0.689 |  |  |
| Forty patients were finally recruited after 2 were excluded because of postoperative death in ICU. *Those covariables were presented as continuous data when entering analyses. ICU, intensive care unit; CI, confidential interval; LVEF, left ventricular ejection fraction; CPB, cardiopulmonary bypass; PDC, peritoneal dialysis catheter; AKI, acute kidney injury; POD, postoperative day. | | | | |
